# Strain Specific Responses in a Microbead Rat Model of Experimental Glaucoma

**DOI:** 10.1080/02713683.2020.1805472

**Published:** 2020-08-25

**Authors:** Karen Eastlake, Hari Jayaram, Joshua Luis, Matthew Hayes, Peng T. Khaw, G. Astrid Limb

**Affiliations:** NIHR Biomedical Research Centre at Moorfields, Eye Hospitaland UCL Institute of Ophthalmology, London, UK

**Keywords:** Glaucoma, electroretinogram, microspheres, intraocular pressure

## Abstract

**Purpose:**

A major challenge in glaucoma research is the lack of reproducible animal models of RGC and optic nerve damage, the characteristic features of this condition. We therefore examined the glaucomatous responses of two different rat strains, the Brown Norway (BN) and Lister Hooded (LH) rats, to high intraocular pressure (IOP) induced by injection of magnetic beads into the anterior chamber.

**Methods:**

Magnetic microsphere suspensions (20 µl of 5–20 mg/ml) were injected into the anterior chamber of BN (n = 9) or LH (N = 15) rats. Animals from each strain were divided into three groups, each receiving a different dose of microspheres. IOP was measured over 4 weeks using a rebound tonometer. Retinal ganglion cell (RGC) damage and function were assessed using scotopic electroretinograms (ERGs), retinal flatmounts and optic nerve histology. ANOVA and Student’s *t*-tests were used to analyse the data.

**Results:**

A significant elevation in IOP was observed in BN rats receiving injections of 20 mg (37.18 ± 12.28 mmHg) or 10 mg microspheres/ml (36.95 ± 13.63 mmHg) when compared with controls (19.63 ± 4.29 mmHg) (*p* < .001) over 2 weeks. This correlated with a significant impairment of RGC function, as determined by scotopic ERG (*p* < .001), reduction in axon number (*p* < .05) and lower RGC density (*P* < .05) in animals receiving 20 mg or 10 mg microspheres/ml as compared with controls. LH rats receiving similar microsphere doses showed reduced scotopic ERG function (*p* < .001) after 2 weeks. No changes in IOP was seen in this strain, although a reduction in axon density was observed in optic nerve cross-sections (*p* < .05). Initial changes in IOP and ERG responses observed in BN rats remained unchanged for a duration of 7 weeks. In LH animals, ERG responses were decreased at 1–2 weeks and returned to control levels after 5 weeks.

**Conclusions:**

Although this model was easily reproducible in BN rats, the phenotype of injury observed in LH rats was very different from that observed in BN animals. We suggest that differences in the glaucomatous response observed in these two strains may be ascribed to anatomical and physiological differences and merits further investigation.

## Introduction

It is well accepted that raised intraocular pressure (IOP) is a risk factor that underlies the onset and progression of retinal ganglion cell (RGC) axon degeneration associated with glaucomatous optic neuropathy,^1^ however, the mechanisms that lead to the pathophysiology of glaucoma are not fully understood. Several experimental models have been used to investigate the molecular and biological mechanisms that lead to IOP-induced optic nerve injury, but they frequently recreate different aspects of the disease.^2^ It is therefore generally recognised that there is not an ideal model that accurately mimics all the features of human glaucoma,^2^ for which much research is still needed. It has been suggested that the strength of each experimental model may depend on its similarity to human disease,^3^ and that due to the differences observed among animal models, the pathophysiological features observed in a given model should not be generalized and ought to be interpreted according to the features of that model.^3^

Current animal models of glaucoma include those that naturally develop, such as the DBA/2 J mouse, as well as experimentally induced models. Although DBA/2 J mice spontaneously develop glaucoma-like features, including increased IOP and RGC loss after 3 months of age,^4^ they show variability in disease progression, and RGC damage may not correlate with increased IOP.^5^ Experimentally induced models of glaucoma have been developed in order to understand the natural history of human disease. Intravitreal injection of N-Methyl-D-Aspartate (NMDA) has been widely used as an established model for rapid RGC damage,^6,7^ although this model has limitations due to excitotoxicity caused by NMDA to other retinal neurons including calretinin ^+^ amacrine cells, and PKCa^+^ bipolar cells, but not rhodopsin^+^ photoreceptors,^8,9^ without causing IOP elevation. Laser photocoagulation has been used to induce scarring in the trabecular meshwork in order to obstruct outflow pathways to induce elevation of IOP and consequently RGC damage.^10^ This procedure has been effectively employed in monkeys, rats and rabbits,^10–12^ but often leads to severe inflammation in the eye.^13^ It also requires the use of expensive equipment and a highly skilled operator.

Microsphere injections to the anterior chamber have been used on a wide variety of animals including primates, mice and rats and have shown robust increases in intraocular pressure and RGC damage.^14–16^ This procedure does not require specialised equipment, making this an ideal model for glaucoma investigations. However, published protocols for this model widely vary in the range of microsphere types and concentrations used, making it difficult for investigators to replicate this method accurately. Using the rat as a model, the majority of investigations using this technique have been conducted predominantly in Brown Norway rats using polystyrene, magnetic or polystyrene ferromagnetic beads ranging between 5 and 15 µm diameter.^15,17–20^ A small number of studies in Sprague Dawley, Albino Swiss and Wistar rats have also been reported in the literature, and also employ a mixture of different size beads (5–10 µm) and types^21–23^ (Supplementary table 1). It is evident that there is a need for a better understanding of this model needed to induce a reproducible increase in IOP and RGC and optic nerve damage, and whether strain differences may be responsible for the variability observed between studies. This is important as commercial availability of certain rat strains may differ between different geographical regions.

This study evaluated the model of high intraocular pressure induced by magnetic microsphere injection into the anterior chamber of the eye in two different rat strains, the Brown Norway rat and the Lister Hooded rat. We investigated the relationship between IOP elevation and retinal ganglion cell damage and axon degeneration in the optic nerve head through functional and histological approaches.

## Methods

### Animals

Wild type Brown Norway and Lister Hooded rats aged 6–9 months were housed under 12-h light/12-h dark cycles and given continuous access to food and water. All animals were maintained according to the U.K. Home Office regulations for the care and use of laboratory animals (Scientific Procedures Act 1986, Licence no. PA021D07F) and adhered to the ARVO Statement for the Use of Animals in Ophthalmic and Vision Research. The use of animals was approved by the local ethics committee at the University College London Institute of Ophthalmology and the U.K. Home Office.

### IOP and magnetic microsphere injections

Baseline intraocular pressure (IOP) measurements were taken prior to bead injection using a rebound tonometer calibrated to the rat eye (Tonolab, Icare). The mean was taken of five readings which were taken during daylight hours in the morning between 10 and 11.30 am. Readings were taken daily whenever possible, excluding weekends. Where appropriate, two-way analysis of variance (ANOVA) with Bonferroni post hoc tests were used for statistical analysis.

Magnetic microspheres (5 µm diameter, BcMag aldehyde terminated magnetic beads; Bioclone Inc) were sterilised by three washes in 70% ethanol, followed by three washes in sterile saline before resuspension to concentrations of 20 mg/ml, 10 mg/ml, or 5 mg/ml for the Brown Norway rat (n = 3 per microsphere dose). Based on our initial studies in the Brown Norway rat, we concluded that 10 mg/ml was optimal to produce a robust glaucoma-like model with sustained rises in IOP and related RGC damage. We used this as a basis to refine the protocol for the Lister Hooded animals, in which we chose to inject microspheres at concentrations of 15 mg/ml, 10 mg/ml and 7.5 mg/ml (n = 5 per microsphere dose).

For the injections, rats were anaesthetised using Ketamine (Narketan; 45 mg/kg) and Domitor (0.375 mg/kg), which was reversed with Antisedan (2.5 mg/kg) after the procedure. Local anaesthesia using oxybuprocaine hydrochloride drops (0.4% w/v Minims; Bausch and Lomb) was also applied. For the microsphere injections, a 30 G needle was used to create a ‘tunnel’ to the anterior chamber to which a Hamilton needle was inserted and 20 µl of microspheres rapidly injected. A magnet (Cube, strength 1.1 kg; Supermagnete, De) used to redirect the microspheres to the iridocorneal angle and a ring magnet (ø6.2 mm; strength 640 g; Supermagnete, De) was placed over the eye for 10 mins before anaesthetic reversal was given (Supplementary Figure 1).

### Scotopic electroretinogram (ERG)

Scotopic electroretinograms (ERGs) were conducted 2 weeks after microsphere injections. Animals were dark adapted overnight and prepared under red light illumination. Animals were anesthetised using ketamine (Narketan; 60 mg/kg) and xylazine (Rompun; 7.5 mg/Kg). Oxybuprocaine hydrochloride (0.4% w/v) drops were used as a topical anaesthetic, and tropicamide (1% w/v, Minims; Bausch and Lomb) and phenylephrine hydrochloride (2.5% w/v, Minims; Bausch and Lomb) drops were used to stimulate pupillary dilation. Once anesthetised, the animal was placed on a heated table under a Ganzfeld stimulator (Diagnosys, UK) housed inside a faraday cage. One subcutaneous ground electrode was placed in the base of the tail, and two reference electrodes were placed in the temporal sides of each eye. Gold wire loop electrodes were placed onto each cornea and lubricated with Viscotears®. Animals were dark adapted for an additional 10 minutes prior to the start of the stimulation protocol.

Scotopic ERGs were recorded using the Espion (Diagnosys) software using protocols previously described.^24^ Briefly, responses were elicited using light flashes ranging from 1.778 × 10^–7^ to 2.0 cd.s.m^−2^ that increased in log steps −0.25 to −0.5. Interflash intervals ranged from 2 to 20 s, with 3–30 recordings per step depending on the light intensity. Responses were filtered using low-frequency cut-off (0.312 Hz) and a high-frequency cut-off (500 Hz). (NB n = 3; LH n = 5 for each microsphere dose)

### ERG analysis

The amplitude of the positive scotopic threshold response (pSTR) was measured from the baseline to the first peak and the negative scotopic threshold response (nSTR) was measured from baseline to the first trough. For comparative analysis of samples, the amplitude of the pSTR and nSTR were measured between −6.75 and −3 log cd.s.m.^2^ The scotopic b-wave amplitudes, measured from baseline to peak, was compared between samples from intensities −5 to −1.5 log cd.s.m.^2^ The scotopic a-wave was measured from baseline to trough and compared between samples from intensities −2 to 1.5 log cd.s.m.^2^ ERG data were analysed using 2-way analysis of variance with Bonferroni post-test corrections.

### Scanning electron microscopy (SEM) examination of the optic nerve head and immunohistological analysis of retinal flat mounts

Optic nerves were fixed overnight in Karnovsky’s fixative (2.5% glutaraldehyde, 2% paraformaldehyde in 0.08 M sodium cacodylate), before washing with phosphate buffer and incubating 2 h in 2% Osmium tetroxide/1.5% Ferricyanide. Samples were then washed in water and placed in 1% thiocarbohydrazide solution for 10 minutes. Another osmication was performed (2% Osmium tetroxide; no FeCn) for 30 mins before incubation in 2% uranyl acetate overnight at 4ºC. Samples were then incubated in Walton’s lead aspartate (pH5.5) at 60°C for 30 mins followed by three washes in water. These were then dehydrated through alcohols (3 min in each solution of 30%, 50%, 70%, 90% and 100% alcohol), placed in acetone 2 × 20 min, and infiltrated with Durcapan resin plus acetone at 25%, 50%, 75% (2 hours each), then 100% overnight, before heating to cure. Ultrathin 1 µm sections were cut (Leica Ultramicrotome) and placed onto acid-washed glass coverslips, then coated with carbon (>10 nm) using an Emitech K950 sputter-coater and mounted onto (scanning electron microscopy) SEM pins with carbon conductive sheets. Edges were sealed with silver paint agar. X5000 magnification images were captured using a backscatter detector on a Zeiss Sigma VP scanning electron microscope and stitched together using the Zeiss SmartStitch software. Optic nerve cross-sections were taken within 1 mm of the optic disc wherever possible. For axon density analysis, axons were counted manually in Image J using 5 representative images (taken in the same locations of all optic nerves, one central and four peripheral equidistant.) in each condition. From this, the axon number per mm^2^ was estimated. Results were analysed using one-way ANOVA with Tukey post-hoc test. (NB n = 3; LH n = 5 for each microsphere dose).

Retinal flat mounts were prepared from tissue of animals perfused with 4% paraformaldehyde (PFA) in phosphate-buffered saline (PBS). Retinae were incubated in blocking buffer (Tris-buffered saline with 5% normal donkey serum; 0.5% triton) for 2 h, followed by addition of primary antibodies and overnight incubation. Retinae were then washed ×3 in tris-buffered saline (TBS) before mounting on glass slides using Flouroshield containing DAPI (Ab104139; Abcam). Primary antibodies used included anti-Neurofilament-200 (1:100; RT.97; Developmental studies Hybridoma bank) and anti-Brn3b (1:50; SC-31984; Santa Cruz Biotech). For analysis, four representative micrograph images were taken of the flat mounts (One in each flatmount quadrant) for each of the animals at 10× magnification. Number of RGC nuclei per mm^2^ were also assessed using the imageJ programme. Results were analysed using one-way ANOVA Tukey post hoc test.

## Results

### Elevation of IOP following microsphere injection into the anterior chamber

**Figure 1.**
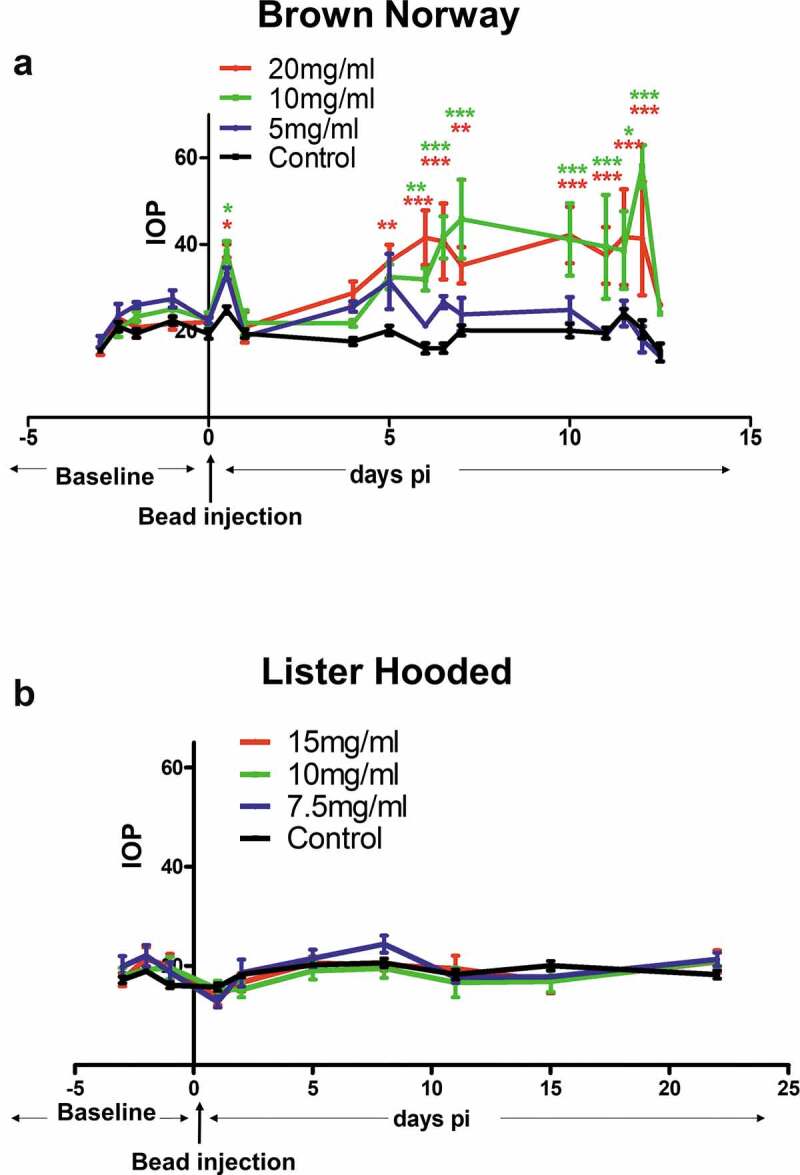
IOP measurements after microsphere injections. Graph (a) shows the difference in IOP levels in the Brown Norway rat between control (untreated) and animals injected with 5 mg/ml (blue line; n = 3), 10 mg/ml (green line; n = 3) and 20 mg/ml (red line; n = 3) microspheres, as assessed over a two-week period. Graph (b) shows the lack of changes in IOP levels in the Lister Hooded rats following injections of 7.5 mg/ml (blue line; n = 5), 10 mg/ml (green line n = 5) and 15 mg/ml (red line; n = 5) microspheres, as compared with control rats. Microsphere injections were conducted at time 0 (*P < .05; **P < .01; ***P < .001)

After microsphere injections into the anterior chamber of the eye showed a significant elevation of the mean IOP in the Brown Norway rats receiving 20 mg microspheres/ml (37.18 ± 12.28 mmHg) (*p* < .001) or 10 mg microspheres/ml (36.95 ± 13.63 mmHg) when compared with (*p* < .001) control eyes (untreated) (19.63 ± 4.29 mmHg) at 6–12 days after injection. There was no difference in the mean IOP between control eyes and eyes receiving 5 mg microspheres/ml (mean IOP 23.76 ± 5.9 mmHg) (Figure 1a). In contrast, Lister Hooded rats followed up for up to 22 days did not show any significant increase in IOP after microsphere injections at all three concentrations tested: (15 mg/ml: 18.68 ± 2.46 mmHg; 10 mg/ml: 17.62 ± 4.36; 5 mg/ml: 19.07 ± 4.76 mmHg; Control: 18.70 ± 3.17 mmHg) (Figure 1b).

### Effect of IOP elevation on the scotopic ERG response

**Figure 2.**
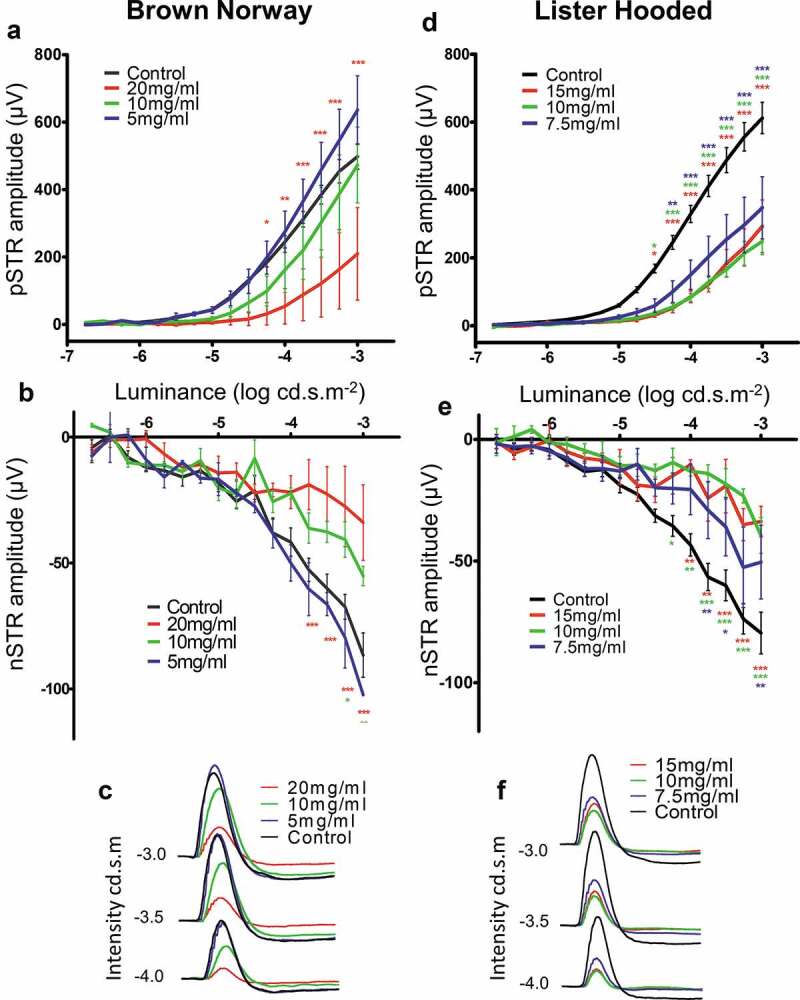
Negative and positive scotopic threshold responses of the ERG after magnetic microsphere injections. Graphs show intensity (log cd.s.m^−2^) and related amplitude (mV) for the pSTR (a,d) and nSTR (b,e) in the Brown Norway (a,b) and Lister Hooded (d,e) rats. Red asterisks indicate significance between control (un-injected fellow eyes) and animals injected with 20 mg/ml or 15 mg/ml microspheres, and green stars indicate significance between control and animals injected with 10 mg/ml microspheres. Blue asterisks indicate significance between controls and animals injected with 7.5 mg/ml microspheres (NB n = 3; LH n = 5 for each experimental condition). (c,f) Example ERG traces for Intensities −3.0, −3.5 and −4.0 log cd.s.m^−2^ for the Brown Norway (C) and Lister Hooded (f) animals, respectively

Scotopic electroretinogram responses were measured 2 weeks after microsphere injections, using the fellow eye as an un-operated internal control. RGC function was assessed by the levels of positive (pSTR) and negative (nSTR) scotopic threshold responses. Brown Norway rats injected with 20 mg microspheres/ml showed a significant reduction in the amplitude of the pSTR for light intensities −4.25 to −3 log cd.s.m^2^ (Figure 2a). No significant differences between control and 10 or 5 mg microspheres/ml for the pSTR were identified. The nSTR analysis revealed that injections of both 20 mg (from −3.75 to – 3 log cd.s.m^2^) and 10 mg microspheres/ml (from −3.25 to −3.0 log cd.s.m^−2^) caused a significant decrease in RGC function (*p* < .001) (Figure 2b). Summation of the responses at light intensities ranging from −4.0 to −3.0 log cd.s.m^2^ for each microsphere dose injected into the Brown Norway rats showed that the overall pSTR and nSTR were clearly impaired after injection of 20 mg or 10 mg microspheres/ml, whilst animals receiving 5 mg microspheres/ml did not show a reduction in the ERG (Figure 2c). Interestingly, in the Lister Hooded rats, a significant reduction (*p* < .001) in the amplitude of the pSTR response was observed at light intensities ranging from −4.5 to −3.0 log cd.s.m^2^ following injection of all microsphere concentrations used (15, 10 and 7.5 mg/ml) (Figure 2d). Similarly, a significant reduction (*p* < .001) in the amplitude of the nSTR at luminance ranging from −4.25 to −3.0 log cd.s.m^2^ was observed after injection of all microsphere concentrations used (15, 10 and 7.5 mg/ml) (Figure 2e). Unlike that seen in the Brown Norway rats, summation of the Lister Hooded rat responses at light intensities ranging from −4.0 to −3.0 log cd.s.m^2^ clearly showed a reduction in both the pSTR and nSTR after injection of any of the three microsphere concentrations (15, 10 and 7.5 mg/ml) used (Figure 2f). No differences were observed in the average pSTR and nSTR of the ERG between the Brown Norway and Lister Hooded control rats (Supplementary Figure 2).

**Figure 3.**
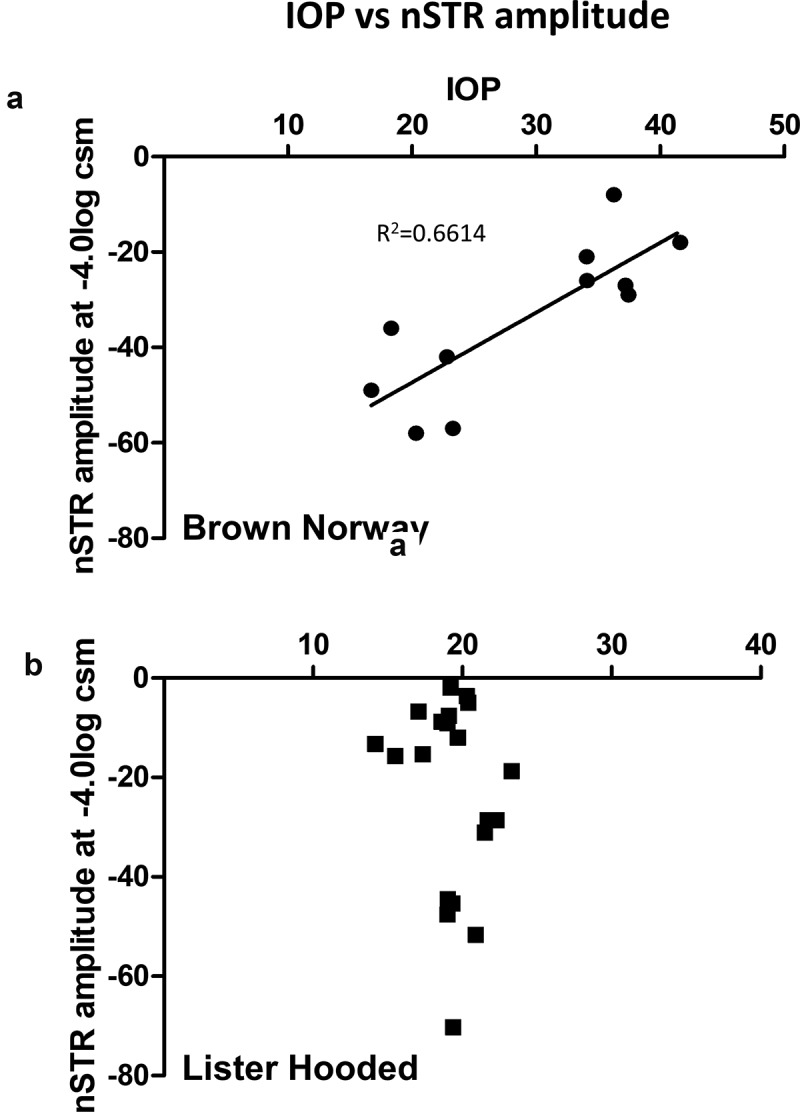
Correlation between IOP and nSTR amplitude. Pearson’s correlation analysis was calculated to evaluate the link between average IOP readings and the amplitude of the nSTR response in the (a) Brown Norway (r^2^= 0.6614, *p* = .0023) and (b) Lister Hooded (r^2^=0.047) rats 1 week after injection of microspheres. (NB n = 3; LH n = 5 for microsphere dose)

A significant correlation (r^2^= 0.6614, *p* = .0023) between the increased IOP average readings and the amplitude of the nSTR response was observed in the Brown Norway rats 1 week after microbead injection (Figure 3a). In contrast, no correlation between the average IOP readings and the nSTR was observed in the Lister Hooded rats (Figure 3b) in which similar IOP was observed after the injection of different microsphere concentrations.

### Evaluation of optic nerve and RGC damage caused by increased IOP

**Figure 4.**
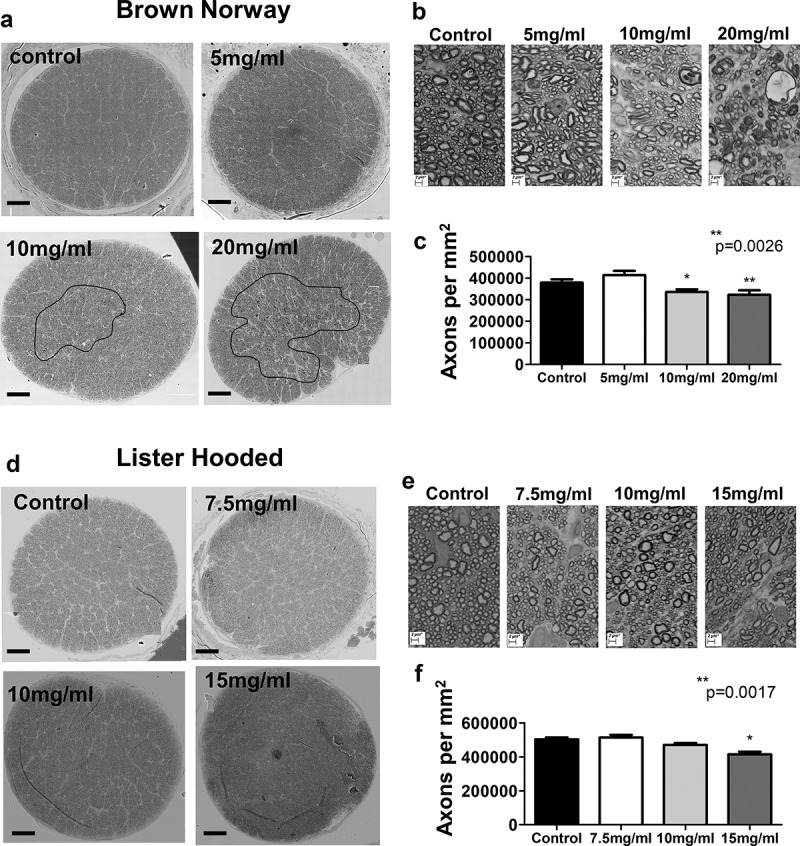
Scanning EM examination of the optic nerve morphology after magnetic microsphere injections. (a) Representative images of optic nerve cross-sections from Brown Norway rats showing damaged areas (highlighted with a circle) under low magnification (x5000 magnification images stitched together using Zeiss SmartStitch) Scale 100 µm. (b) Higher magnification images (x5000 magnification) of axon and myelin morphology in Brown Norway rats receiving none (control), 5 mg/ml, 10 mg/ml and 20 mg/ml microspheres. (c) Bar graphs show the estimated axon counts per mm^2^ in Brown Norway rats between controls and animals injected with 5 mg/ml, 10 mg/ml and 20 mg/ml microspheres (**P = .0026, one-way ANOVA with Tukey post-test). (d) Representative images of optic nerve cross-sections from Lister Hooded rats showing no obvious damaged areas under low magnification (5000× magnification images stitched together using Zeiss SmartStitch). Scale 100 µm (e) Higher magnification images (5000× magnification) of axon and myelin morphology show no morphological changes in the optic nerve head of Lister Hooded animals receiving 7.5 mg/ml, 10 mg/ml and 15 mg/ml microspheres as compared with the controls. (f) Bar graphs show differences in the estimated axon density per mm^2^ in Lister Hooded rats between controls and animals injected with 7.5 mg/ml, 10 mg/ml or 15 mg/ml microspheres (**P = .0017, one-way ANOVA with Tukey post-test). (NB n = 3; LH n = 5 for each microsphere dose)

Examination of optic nerve cross sections by SEM in Brown Norway rat eyes showed clear areas of axonal damage in rats receiving 20 mg and 10 mg microspheres/ml into the anterior chamber. Axonal damage was not evident in animals injected with 5 mg microspheres/ml when compared with control non-injected eyes in low magnification images (Figure 4a). High magnification (5000x) images of the optic nerve in Brown Norway rats showed damage to axons, with axonal swelling and myelin debris, in eyes injected with 10 mg or 20 mg microspheres/ml (Figure 4b). There was no difference between the axon density of control animals and those that received 5 mg microscpheres/ml. A significant reduction in the axon number estimated to per mm^2^ was found between 5 mg/ml and 20 mg/ml, and 10 mg/ml in these rats, indicating the severity of the optic nerve damage (Figure 4c). In contrast, axonal damage was not obviously striking in the optic nerve images of Lister Hooded rats (Figure 4d). Very little to no axonal swelling or myelin damage could be seen in cross sections of optic nerve head from eyes injected with any of the three concentrations of microspheres used (Figure 4d and e). However, analysis of higher magnification images (x5000) of optic nerve sections in this rat strain showed that the axon numbers estimated per mm^2^ were significantly reduced in animals receiving 15 mg/ml microspheres when compared with the control eyes (Figure 4f).

**Figure 5.**
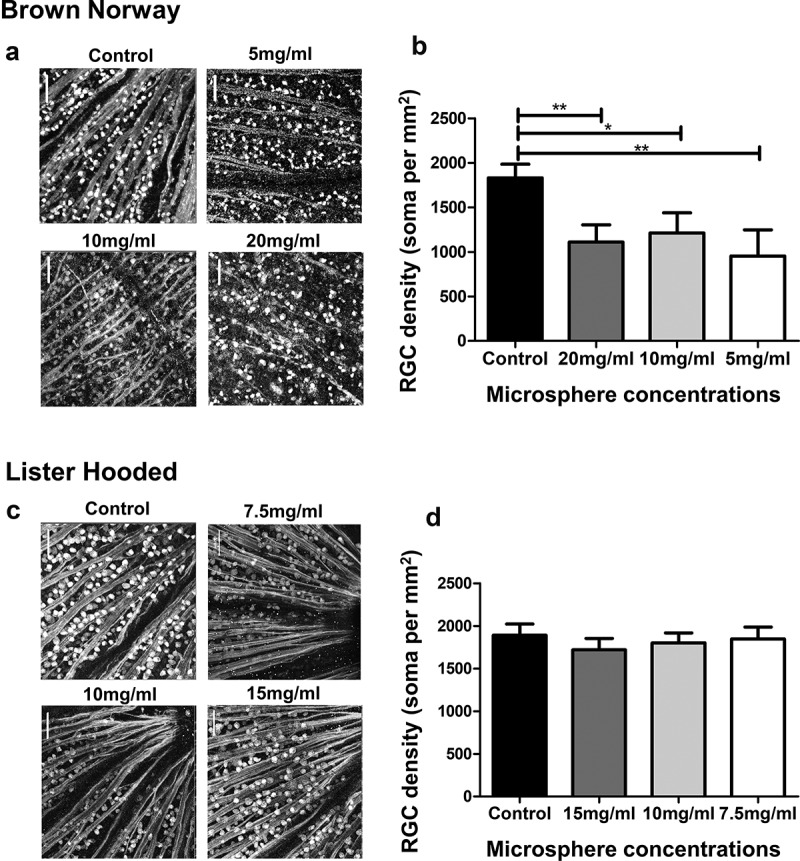
Examination of nerve fibre and RGC soma damage. Images show immunofluorescence staining of retinal flat mounts for Brown Norway (a) and Lister Hooded (c) animals. RGC Nuclei are stained with antibodies to Brn3a and nerve fibres with antibodies to neurofilament-200. (b) Histogram shows differences in RGC density in Brown Norway rats between controls and animals injected with 20 mg/ml, 10 mg/ml and 5 mg/ml microspheres (*P < .05, **P < .01). (d) Histogram shows the lack of differences in RGC density in Lister Hooded rats between controls and animals injected with 15 mg/ml, 10 mg/ml and 7.5 mg/ml microspheres. RGC density was calculated by dividing the soma count by the area. (NB n = 3; LH n = 5 for each microsphere dose)

Microscopic examination of retinal flat mounts to assess RGC density (cells per mm^2^) and nerve fibre damage revealed damage to RGC fibres in the Brown Norway rat retina after injection of 20 mg or 10 mg microspheres/ml into the anterior chamber (Figure 5a). RGC density, expressed as the number of cells per mm^2^, showed a significant reduction in the Brown Norway retinae receiving 20, 10 or 5 mg microspheres/ml as compared to controls (Figure 5b). In comparison, flat retinal mounts from Lister Hooded rats did not show any apparent damage to the RGC fibres (Figure 5c). This was reflected in the number of RGC density, which showed no significant reduction in the number of RGC per mm^2^ for all the three microsphere concentrations tested (5, 10 and 7,5 mg/ml) (Figure 5d).

### Long term analysis of RGC function after microsphere injection

**Figure 6.**
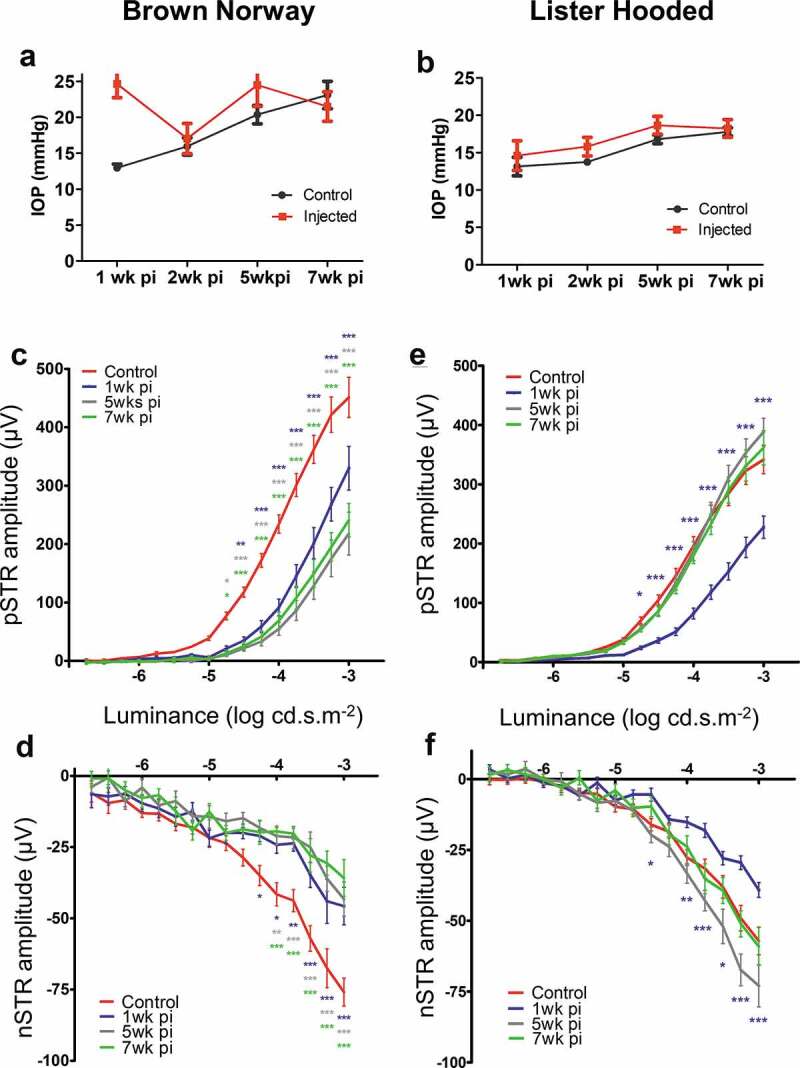
Long term assessment of IOP after microsphere injections into the anterior chamber (a-b) Graphs shows the difference in IOP levels in the Brown Norway (a) and Lister hooded (b) rat between control (untreated) and animals injected with 10 mg/ml microspheres, at 1, 5 and 7 weeks after injection. Graphs (c–f) show intensity (log cd.s.m^−2^) and related amplitude (µV) for pSTR (c,e) and nSTR (d,f) in the Brown Norway (c,d) and Lister Hooded (e,f) rats. Asterisks indicate significance between control (red) and treated animals at 1 (blue), 5 (grey) or 7(green) weeks after microsphere injection (*P < .05; **P < .01; ***P < .001). (Brown Norway n = 8; Lister Hooded n = 12)

Since injection of 10 mg/ml microspheres provided an optimal rise in IOP and sufficient damage to the RGCs in Brown Norway rats (shown above), to further examine implications on IOP and RGC function, a separate group of Brown Norway and Lister Hooded animals were injected with 10 mg/ml microspheres and analysed over a period of 7 weeks. One week after injection, the IOP in the Brown Norway rat was elevated to an average of 24.65 ± 5.3 mmHg as compared to 12.9 ± 1.4 mmHg in the fellow uninjected eye (Figure 6a). This IOP elevation persisted over the 7 week period of the study (Figure 6a). We also observed a gradual increase in the IOP of the fellow un-injected eye over the course of the 7 week study in the BN rat (Figure 6a). In contrast, IOP in the Lister hooded rats was not significantly raised in the injected or the fellow control eye over the 7 week duration of the study (Figure 6b).

Scotopic ERG responses measured in the Brown Norway rats at 1 week after microsphere injections showed a significant decrease in the pSTR response at light intensities −4.75 to −3.0 log cd.s.m^−,2^ when compared with the un-injected fellow eye (ANOVA with Bonferonni post hoc test; *p* < .0001). This decrease was sustained at 5 and 7 weeks after bead injection, without any further significant decrease observed (Figure 6c). Similarly, a significant impairment in the nSTR was observed at light intensities −4.5 to −3.0 log cd.s.m^−2^ at 1 week after injection. This response persisted for the 7 week duration of the study (Figure 6d). Analysis of the pSTR at light intensities −4.75 to −3.0 log cd.s.m^−2^ in the Lister hooded rats also showed a significant reduction at 1-week post injection (Figure 6e); however, the nSTR levels had returned to those of the control eyes at 5–7 weeks after microbead injection. A similar pattern was observed when recording the nSTR at 1-week post microsphere injection, when a significant impairment to the nSTR measured at −4.5 to −3.0 log cd.s.m^−2^ was observed, but was followed by a recovery to control levels at 7 weeks (Figure 6f).

## Discussion

Animal models that closely mimic the pathological features of glaucoma are needed for effective investigations into the disease aetiology and therapeutics. In this study, we assessed a method frequently used to increase IOP by injection of magnetic microspheres into the anterior chamber. Reports in the literature regarding this method have used a wide range of microsphere types in rodents, yet, there is no clear reproducibility of the model and the extent of RGC and optic nerve damage widely differs amongst publications (Supplementary Table 1). On this basis, we have analysed the reproducibility of this method using 5 µm diameter magnetic microspheres at different concentrations in two rat strains, Brown Norway and Lister Hooded rats.

The significant increase in the mean IOP of Brown Norway rats observed in this study upon injection of 20 mg and 10 mg microspheres/ml is in accordance with previous studies using 5 µm magnetic microspheres in this strain.^19,22^ However, unlike previous studies which have reported a rapid increase in IOP that slowly reduces over time,^23^ we observed a gradual increase in IOP after 2 days, that was maintained for the 2-week duration of the initial study and for the 7 week period of a subsequent study.

In contrast, we did not observe any increase in the IOP of Lister Hooded rats after microsphere injections over a 7-week study. Since IOP was measured once a day during light hours, it may be possible that we missed a nocturnal rise in IOP that may have impaired their RGC function; therefore, it would be important to conduct frequent measurements of IOP to reveal any patterns in IOP fluctuations in this strain. This has been suggested by other studies investigating aqueous humor outflow and circadian rhythms after episcleral vein occlusion, that show that IOP is more likely to increase in the dark phase rather than in the light,^25^ which might account for some of the differences in our study. Interestingly, although we observed a reduction in axon density in optic nerve cross sections of the lister hooded rats, this did not correlate with the number of RGC soma counts in retinal flatmounts. The observation that the axon number in the optic nerve of the LH rat was significantly reduced but that no impairment to RGC was observed, suggests either mild damage to axons that was not reflected in soma numbers or that the experimental methods used were not sensitive enough to detect any changes. It would be important to further investigate anatomical and physiological differences between these two strains, which may prevent Lister Hooded rats from developing obvious glaucomatous features upon injection of microbeads into the anterior chamber.

Although previous studies have shown glaucoma-like damage in different strains, including Wistar, Sprague Dawley and Swiss Albino rats,^21–23^ our results suggest that this model cannot be accurately reproduced in the Lister hooded rat, and significant adaptations to the protocol may be required. This is also mirrored when comparing published protocols from the literature in several rat strains. Various groups have utilised different bead materials (polystyrene/magnetic/latex), sizes (5–15um), volumes (2.5–25ul) and concentrations (Supplementary Table 1), which can be problematic when selecting a strain and protocol for glaucoma investigations.

We assessed the level of functional damage in the retina caused by increased IOP and observed that Brown Norway rats receiving 20 mg or 10 mg microspheres/ml showed a significant reduction in the pSTR and nSTR. In this strain, increased IOP was closely related to the decrease in RGC function as judged by the scotopic ERG. This was confirmed by the loss of RGC soma and axonal damage as assessed by microscopical examination of the retina and optic nerve. Although no IOP increase was observed in the Lister Hooded animals, impairment to the pSTR and nSTR was observed after 1 week when similar microsphere concentrations to those used in the Brown Norway were injected into this strain. As previously noted, it is possible that the IOP readings taken did not capture the rise in IOP due to the time of the day in which the readings were made or that the equipment used may not have been sensitive enough to detect any minor changes occurring the LH animals. We also observed that in Lister Hooded animals, but not the Brown Norway rats, administration of phenylephrine and tropicamide drops to the microsphere-injected eye, did not induce complete dilation of the pupil, which may have affected the ERG recordings. However, this lack of pupil dilation cannot completely account for the significant impairment of the RGC responses observed. It has been previously noted that anatomy and cell density in the lamina cribrosa can vary between rat strains, which may impact in the development of experimental models of glaucoma.^26^ It is possible that the differences between the two rat strains observed in this study may be due to either anatomical differences in the lamina cribrosa or the 3D structure of the trabecular meshwork, and this would be important to investigate before using different rat strains to induce this experimental model.

Over the course of a 7 week study using 10 mg microsphere/ml injection into the anterior chamber of the Brown Norway rat, we observed a gradual increase in the IOP of the fellow un-injected eye, whereas the IOP in the injected eyes remained relatively constant. These findings have been previously reported in a different model of experimental glaucoma in the rat^27^ and may suggest an adaptation to the rise in pressure of the injected eye or the circulation of inflammatory factors throughout the optic tract that may eventually affect the fellow eye. It is important that damage to the retinal function remained persistent over 7 weeks, suggesting that this model represents the neural damage observed in glaucoma. More interestingly, the impairment in the ERG response observed in the Lister hooded animals at 2 weeks after microsphere injection, did not persist at 5 or 7 weeks post injection and indicated that function had returned to control levels. It is possible that the microspheres injected in the LH rats induced a very mild impairment to the RGC function as seen by the reduction in scotopic ERG responses observed at weeks 1–2, which allowed for a recovery in function after 5–7 weeks. This was in agreement with the inconsistency observed between the axon number and RGC counts of LH rats, where methods may not have been sufficiently sensitive to detect very minor changes. As previously suggested there are likely to be anatomical differences between strains that account for responses observed, which will be important to address in future studies.

## Conclusion

We conclude that the microbead model of experimental glaucoma is highly reproducible and easy to conduct in Brown Norway rats. However, this model was not reproduced when using the Lister Hooded rat strain, suggesting that this model induces different responses in various rat strains, which explains the wide variability of data reported by other experimental studies utilizing this model. Specific responses may need to be taken into consideration when selecting animal models for glaucoma studies, and more importantly, when attempting to investigate the effect of potential treatments on this condition. Further investigations on the physiological and anatomical differences between strains may aid in the selection of animal models for experimental studies of glaucoma therapies.

## Supplementary Material

Supplemental Material
