# Supplementary material for: Strain Specific Responses in a Microbead Rat Model of Experimental Glaucoma
Source: Curr Eye Res. 2020 Aug 25;46(3):387–97. doi: 10.1080/02713683.2020.1805472 (PMC8025805; doi:10.1080/02713683.2020.1805472)
Supplement: Supplemental Material [file ICEY_A_1805472_SM9670.zip › Supplementary Table.pdf]

Supplementary table 1: Microbead Model differences reported in the literature.

**Author**	**Strain**	**Age/Weight**	**Bead type**	**Size**	**Volume**	**Concentration/ Bead No.**	**Duration of IOP elevation**	**Peak IOP (mmHg)**	**Damage assessment**
Abbott 2014	BN	203-226g 2-3month M /330-397g ex-breeders 9.5-10.5 months M	Magnetic	10um	10ul (5ul for 2nd or 3rd injection)	30mg/ml	3wks ; (1-3 repeat injections)	61.2+-6.2 (2month) 61.9+-12.1(9-month)	Retinal nerve fibre layer thinning – 7.4+-11.8% (2month) 21.4-+7.6%(9month)
Bunker 2015	BN	250g-300g ex-breeder F	Magnetic	8um	25ul	30mg/ml	18days	49.9+-2.3	Tunel assay – 15 fold increase in apoptotic RGC nuclei
Foxton 2013	BN	250g-300g ex-breeder F	Polystyrene ferromagnetic	8um	25ul	30mg/ml	17days	55.2+-3.5	Tunel Assay – 16-fold increase in apoptotic RGC nuclei
Samsel 2011	BN	Ex-breeder M	Magnetic	5um	10-20ul	0.3-0.6mg beads delivered (30mg/ml)	12days	35.8+-1.2	RGC density count – 36.4-+2.4% loss in RGC
Sappington 2010	BN	3-7 months	Polystyrene	15um	2.5ul/5ul/7ul	1x10^6 microbeads /ml	2wks, 8wks reinjected	32	16% lower axon density
Urcola 2006	SD, Albino; F	250-300g	Latex microspheres	10um	20ul	2-4x10^5 injected	30wks; weekly injections (9 injections total)	37.6+-2.6	RGC soma density analysis – 23.1+_2.1% RGC death
Dai 2012	Albino Swiss		Polystyrene ferromagnetic	5um	20ul	30mg/ml	4wks	43	81% axon loss after 4wks
Smedowski 2014	Wistar	42wks old	polystyrene	10+6um mix	15ul (5ul; viscoat ; 5ul 6um + 5ul 10um)	-	6wks	43.7	34.6% loss of RGC

Supplementary table 1: Microbead Model differences reported in the literature.

Urcola 2006 J.H. Urcola, M. Hernández, E. Vecino **Three experimental glaucoma models in rats: comparison of the effects of intraocular pressure elevation on 18 retinal ganglion cell size and death**
Exp. Eye Res., 83 (2) (2006), pp. 429-437

A. Smedowski, M. Pietrucha-Dutczak, K. Kaarniranta, J. Lewin-Kowalik **A Rat Experimental Model of Glaucoma Incorporating Rapid-onset Elevation of Intraocular Pressure**
Sci. Rep., 4 (2014), p. 5910, 10.1038/srep05910

R.M. Sappington, B.J. Carlson, S.D. Crish, D.J. Calkins **The microbead occlusion model: a paradigm for induced ocular hypertension in rats and mice**
Invest Ophthalmol Vis Sci, 51 (1) (2010), pp. 207-216

P.A. Samsel, L. Kisiswa, J.T. Erichsen, S.D. Cross, J.E. Morgan **A novel method for the induction of experimental glaucoma using magnetic micro-spheres**
Invest Ophthalmol Vis Sci., 52 (3) (2011), pp. 1671-1675

R.H. Foxton, A. Finkelstein, S. Vijay, A. Dahlmann-Noor, P.T. Khaw, J.E.Morgan, *et al.* **VEGF-A is necessary and sufficient for retinal neuroprotection in models of experimental glaucoma**
Am. J. Pathol., 182 (4) (2013 Apr), pp. 1379-1390

S. Bunker, J. Holeniewska, S. Vijay, A. Dahlmann-Noor, P. Khaw, Y.-S. Ng, *et al.*
J. Vis. Exp., 96 (2015), p. e52400, 10.3791/52400

C. Dai, P.T. Khaw, Z.Q. Yin, D. Li, G. Raisman, Y. Li **Structural basis of glaucoma: the fortified astrocytes of the optic nerve head are the target of raised intraocular pressure**
Glia, 60 (1) (2012), pp. 13-28

Supplementary table 1: Microbead Model differences reported in the literature.

Abbott CJ, Choe TE, Burgoyne CF, Cull G, Wang L, Fortune B (2014) Comparison of Retinal Nerve Fiber Layer Thickness In Vivo and Axonal Transport after Chronic Intraocular Pressure Elevation in Young versus Older Rats. PLoS ONE 9(12): e114546. https://doi.org/10.1371/journal.pone.0114546
