# Supplementary figures and images for: Strain Specific Responses in a Microbead Rat Model of Experimental Glaucoma
Source: Curr Eye Res. 2020 Aug 25;46(3):387–97. doi: 10.1080/02713683.2020.1805472 (PMC8025805; doi:10.1080/02713683.2020.1805472)

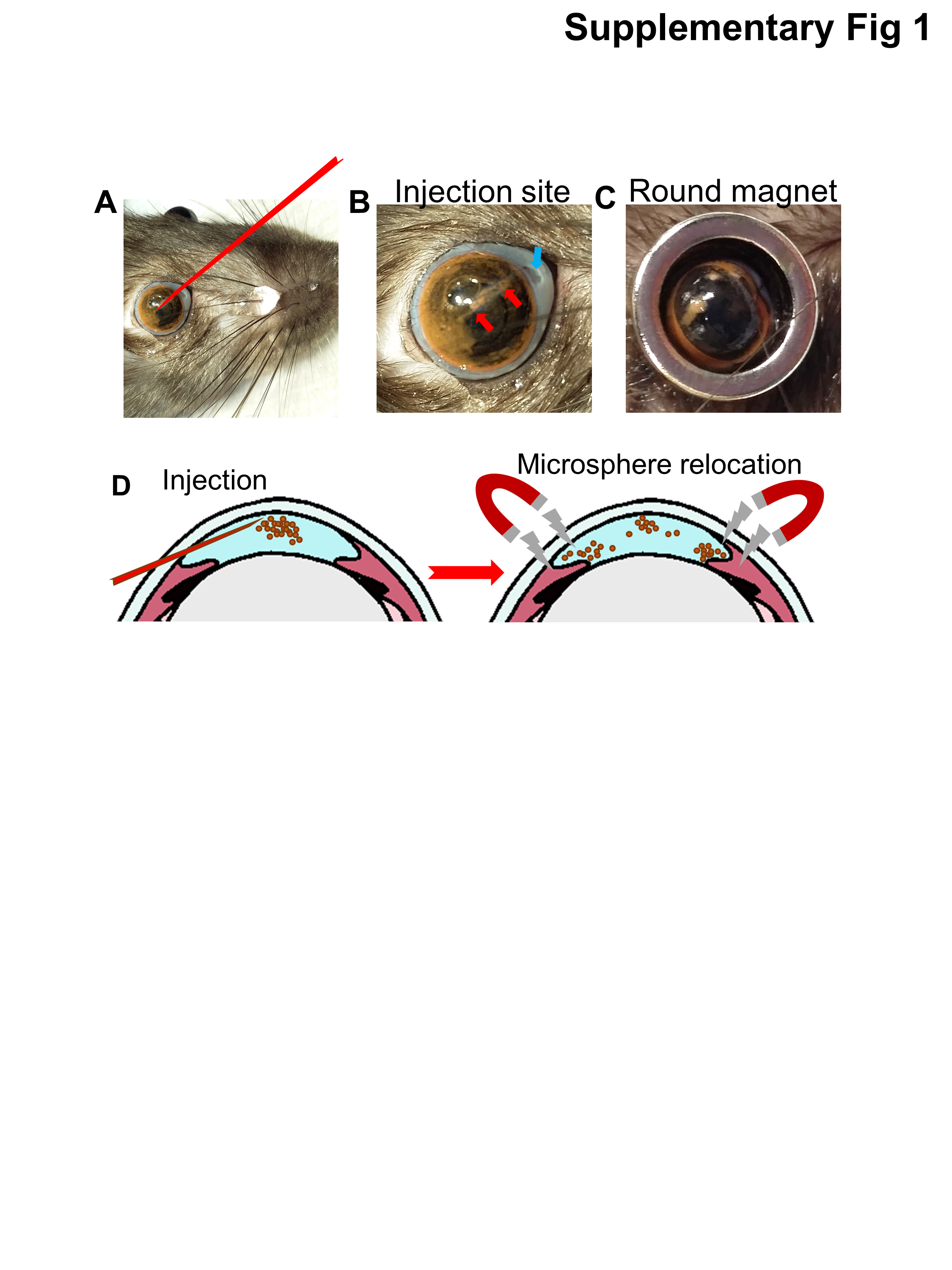

Supplement: Supplemental Material [file ICEY_A_1805472_SM9670.zip › ncer-2020-or-0606-File008.tif]

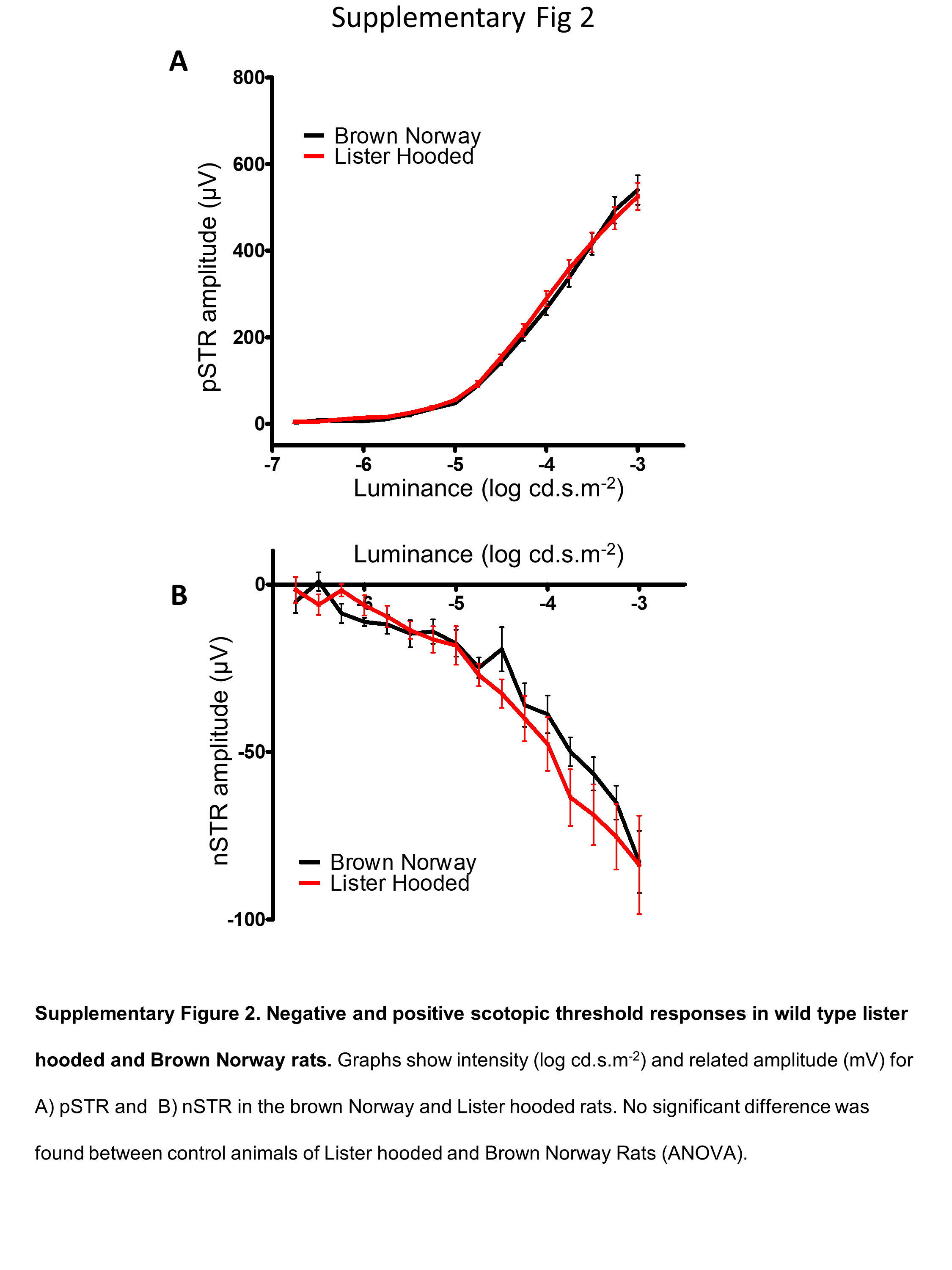

Supplement: Supplemental Material [file ICEY_A_1805472_SM9670.zip › ncer-2020-or-0606-File009.tif]
